# Life History of the Giant Looper Moth *Ascotis selenaria* (Lepidoptera: Geometridae) in Eucalyptus Plantations and the Effect of Adult Mating Age on Fecundity

**DOI:** 10.3390/biology14121780

**Published:** 2025-12-13

**Authors:** Shuai Yuan, Mengjun Yang, Rijiao He, Bin Liu, Sijia Wang, Zhende Yang, Ping Hu

**Affiliations:** Guangxi Colleges and Universities Key Laboratory for Cultivation and Utilization of Subtropical Forest Plantation, Guangxi Key Laboratory of Forest Ecology and Conservation, College of Forestry, Guangxi University, Nanning 530004, Chinahrijiao@163.com (R.H.);

**Keywords:** *Ascotis selenaria*, eucalypt, biological characteristics, reproductive biology, Integrated Pest Management (IPM)

## Abstract

*Ascotis selenaria* has recently shifted hosts to become a major defoliator in Southern China’s eucalyptus plantations. To facilitate Integrated Pest Management (IPM), we investigated the genetic origins, life history, and reproductive bio-ecology of this population. Mitochondrial COI analysis revealed that the Southern China population aligns phylogenetically with South Asian clades, distinct from Northern China populations. Life table analysis confirmed six larval instars, with the final instar exhibiting exponential consumption, accounting for 79.68% of total food intake. Reproductive assays demonstrated significant protandry and a novel bimodal ovarian maturation rhythm (peaking on days 3 and 7). Crucially, female fecundity declined sharply after a 3-day mating delay, and mating with older males severely reduced egg hatchability in older females. These findings suggest that control thresholds must shift from visual damage assessment to monitoring early-instar larvae (1st–3rd instars). Furthermore, the combination of protandry and reproductive sensitivity implies that mating disruption strategies must be deployed prior to male emergence. This study provides the biological basis for a dual-window IPM framework targeting this emerging pest.

## Simple Summary

The Giant Looper moth (*Ascotis selenaria*) has recently been detected causing significant defoliation in Eucalyptus plantations in Southern China. To improve management strategies, we investigated its genetic origins, life cycle, and reproductive biology on this host. Mitochondrial COI analysis showed that the Southern China population is genetically closely related to populations in South Asia (Pakistan/India). Life table analysis showed that larvae have six instars, with the final three instars accounting for over 98% of total food consumption. Regarding reproduction, males emerge 1–2 days before females (protandry). Females are highly sensitive to mating delays; failure to mate within 3 days of emergence significantly reduces fecundity. We also observed a unique “two-peak” pattern in ovarian maturation. These findings indicate that pest control interventions should target early-instar larvae (1st–3rd instars) before peak consumption occurs.

## 1. Introduction

The eucalyptus industry holds a critical position in the economic development of Southern China [1]. However, the ever-increasing scale of artificial afforestation, marked by extensive and intensive monoculture of this single tree species, has given rise to multiple ecological challenges. Among these, a reduction in biodiversity has led to the outbreak of various pests and diseases [2]. Damage caused by geometrid moths, characterized by short generation times and high reproductive capacity, is particularly severe, establishing them as one of the most difficult groups of eucalypt defoliators to control [3]. This pattern has been confirmed globally: in Brazil, the geometrid *Thyrinteina arnobia* is a major eucalypt pest [4]; and in neighboring Vietnam, *Biston suppressaria* (another geometrid) has also become a serious threat [5]. Consequently, the recent detection of defoliation by *Ascotis selenaria* (Lepidoptera: Geometridae) in Southern China’s eucalypt forests is consistent with a global trend of adaptive evolution in geometrid pests, representing a significant concern. Currently, control of this pest in eucalyptus plantations relies primarily on the application of broad-spectrum chemical insecticides. However, this approach often leads to environmental pollution and the disruption of natural enemy populations, highlighting the need for more specific biological and ecological control measures.

*A. selenaria* is a known severe pest with a wide geographical distribution spanning Asia, Africa, and Europe [6]. Its most notable characteristic is its high polyphagy, which allows it to cause serious economic damage to various high-value crops. Literature explicitly documents it as a major pest of coffee in Africa [7], avocado in Israel [6], citrus in Korea [8], and tea in India [7]. The potent host adaptiveness exhibited by *A. selenaria* indicates that it is a highly opportunistic pest, capable of rapidly exploiting the novel and abundant food resource provided by the large-scale eucalypt monocultures in China.

Given its economic importance, *A. selenaria* has been extensively studied in other host systems. For example, researchers in Korea have developed a temperature-dependent developmental model on citrus [9]; while studies in Israel have addressed its life cycle, monitoring techniques, and biological control on avocado [6,10]. However, a critical knowledge gap remains: the life history and demographic parameters of polyphagous insects are highly dependent on the specific host plant consumed [11]. Previous studies have shown that *A. selenaria* exhibits plasticity in developmental time and fecundity when feeding on different crops like citrus or tea. However, data obtained from citrus or avocado cannot be directly extrapolated to predict its population dynamics or to formulate control thresholds on eucalyptus. Currently, there is a severe global lack of basic biological data for *A. selenaria* specifically on the eucalypt host system.

To minimize ecological disruption while protecting this essential forestry timber industry, the development of sustainable Integrated Pest Management (IPM) strategies is paramount [12]. Any effective IPM program must be founded upon a deep, specific understanding of the target pest’s life cycle, ecological habits, and behavioral patterns [13]. Current global research on *A. selenaria* primarily focuses on its fundamental biological characteristics, and although these studies provide a foundational framework for understanding its basic ecology, differences in regional climatic conditions render them insufficient for formulating specific field management strategies and control measures in Southern China. In contrast, studies on several other pests have yielded significant advances, facilitating in-depth research and large-scale rearing, yet similar research on *A. selenaria* is scarce. This study focused on *A. selenaria* infesting eucalyptus, using the natural host plant for rearing. We combined molecular and morphological identification to clarify its taxonomic status, detailed its biological characteristics, and conducted experiments on mating and oviposition across different adult ages to determine reproductive capacity. The results will provide a theoretical basis for subsequent in-depth research on the feeding mechanisms and regulatory characteristics of this insect, offering the necessary biological foundation for formulating science-based IPM strategies against this emerging pest.

## 2. Materials and Methods

### 2.1. Insect Rearing

The *A. selenaria* larvae used for the experiments were collected from Sanmenjiang State Forest Farm in Liuzhou, Guangxi. The collected larvae were reared in the laboratory using leaves of *Eucalyptus urophylla*. Late-instar larvae were transferred to Petri dishes upon the onset of pupation. Newly emerged adults were immediately moved to mating cages, where an oviposition board was placed for egg-laying by females. A 10% honey–water solution was provided to maintain adult vitality. Eggs were stored in culture dishes until hatching. Fifteen newly hatched larvae were randomly selected and placed individually into 1.5 mL centrifuge tubes. The tube bottoms were perforated and plugged with moist absorbent cotton to keep the eucalypt leaves fresh. When the larvae attained sufficient size, they were transferred to Petri dishes for individual rearing, with a half-sheet of moist filter paper (Φ 6 cm) used to maintain humidity. Larval feeding and molting were observed daily; shed head capsules were collected, leaves were replaced, and environmental hygiene was maintained. Rearing conditions were maintained at a temperature of (26 ± 2) °C, a photoperiod of L:D = 14:10 h, and a relative humidity of 75 ± 10%. Sex identification was performed following methods described in previous studies [8,14], based on the antennal morphology for adults and the position of the genital opening for pupae.

### 2.2. Morphological and Molecular Identification

Identification was performed using a combination of morphological analysis and DNA barcoding. Specimens of all developmental stages (egg, larva, pupa, adult) were collected and their morphological characteristics were observed and recorded under a stereomicroscope (Olympus SZX16, Olympus, Tokyo, Japan). Species identification was conducted based on morphological descriptions and taxonomic keys provided by Han [15].

Total DNA was extracted from adult leg tissue using the EasyPure^®^ Genomic DNA Kit (TransGen Biotech Co., Ltd., Beijing, China). Polymerase Chain Reaction (PCR) amplification was performed using the universal insect mitochondrial COI gene primers LCO-1490 (5′-GGTCAACAAATCATAAAGATATTGG-3′) and HCO-2198 (5′-TAAACTTCAGGGTGACCAAAAAATCA-3′). The PCR system (25 μL) and amplification conditions were set as follows: initial denaturation at 94 °C for 2 min; followed by 30 cycles of 94 °C for 30 s, 55 °C for 30 s, and 72 °C for 30 s; and a final extension at 72 °C for 2 min. PCR products were sequenced and subjected to BLAST comparison (https://blast.ncbi.nlm.nih.gov/, accessed on 19 November 2025) in GenBank. Simultaneously, COI sequences of Ascotis species and closely related species were downloaded, and a phylogenetic tree was constructed using the Neighbor-Joining (NJ) method in MEGA 11 software, with bootstrap repetitions set to 1000.

### 2.3. Study of Life History and Developmental Characteristics

#### 2.3.1. Generation Duration and Annual Life History

To measure the generation duration, 15 newly hatched larvae were randomly selected and reared individually in Petri dishes. The duration of the egg stage, each larval instar, pre-pupal stage, pupal stage, and adult longevity were observed and recorded daily. To obtain more stable data for the pupal and adult stages, a separate centralized rearing cohort (*n* = 228) was established, and their pupal duration and adult longevity were statistically analyzed, along with the total number of generations per year.

#### 2.3.2. Larval Instar Division, Morphological Measurement, and Feeding Analysis

During the individual rearing described in Section 2.3.1, shed larval head capsules were collected daily. The head capsule width was measured using a stereomicroscope, and instars were divided based on Dyar’s law [16]. Larval body length was also recorded. To measure the feeding volume, fresh eucalypt leaves were provided daily to the individually reared larvae. Before replacement and after 24 h of feeding, the leaves were scanned using an EPSON 12000XL scanner (EPSON, Los Alamitos, CA, USA), and the area of the leaf consumed (cm^2^) was analyzed using WinRHIZO Pro 2019a. The daily feeding volume (g) of the larvae was then converted using the pre-determined average dry weight per unit leaf area (g/cm^2^).

### 2.4. Adult Reproductive Biology Study

#### 2.4.1. Dissection of Male and Female Reproductive Systems

Female and male adults of different ages (1–9 days old) and before and after mating (within 12 h post-mating) were dissected under (26 ± 2) °C conditions. Dissections were performed in Ringer’s physiological saline. Key indicators, such as the length of the female ovarioles, the developmental status of oocytes, and the size of the male testes and the morphology of the single ejaculatory duct, were observed, measured, and photographed using a stereomicroscope.

#### 2.4.2. Relationship Between Ovarian Development and Adult Age

Based on the dissection results, the ovariole length of female moths aged 1–9 days was measured. Simultaneously, the number of fully mature eggs (green) and immature oocytes (yellow-green) within the ovarioles was counted. The degree of egg maturation was calculated as the ratio of the number of mature eggs to the total number of eggs in the ovarioles.

#### 2.4.3. Experimental Design for Reproductive Capacity

Adults reared individually (one female and one male) after emergence were divided into three age groups: 1–3 days, 4–6 days, and 7–9 days. Pairings were conducted according to a 3 × 3 experimental design. To increase the mating rate, a sex ratio of 1♀:2♂ was used, and the individuals were placed in a single mating cup. Each male moth was used only once, and each experimental group was replicated 3–6 times. The total number of eggs laid by the female moths in each group was counted, and a random egg mass was selected to determine the hatchability.

### 2.5. Statistical Analysis

All experimental data were statistically analyzed using IBM SPSS Statistics 25.0 software and plotted using OriginLab 2021. Quantitative data are presented as Mean ± Standard Error (SE). Prior to analysis, data were checked for normality using the Shapiro–Wilk test and for homogeneity of variances using Levene’s test. Independent samples t-tests were used for comparison between two groups (e.g., female and male pupal duration, longevity). One-Way Analysis of Variance (ANOVA) was employed for multiple-group comparisons (e.g., head capsule width across different instars). For the effect of adult age on reproductive capacity, Two-Way ANOVA was used to test the main effects of female age, male age, and their interaction effect. Duncan’s New Multiple Range Test (DMRT) was used for all post hoc multiple comparisons. A value of *p* < 0.05 was considered statistically significant.

## 3. Results

### 3.1. Taxonomic Identification of Species

**Figure 1:**
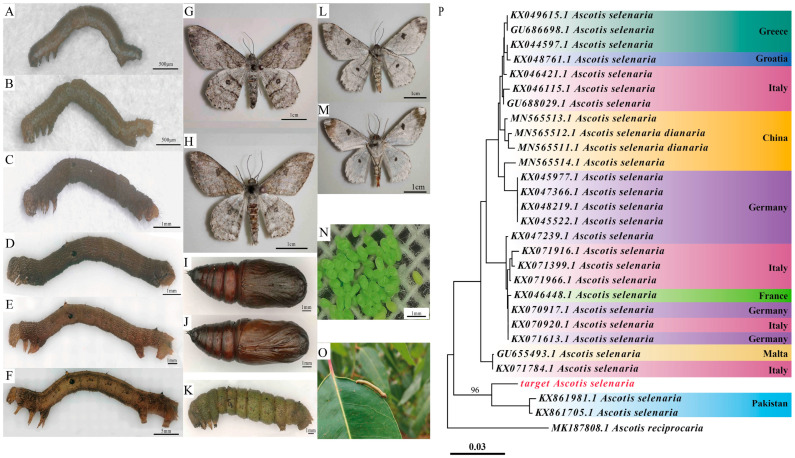
Morphology of *A*. *selenaria* and the Neighbor-Joining tree based on COI gene barcode sequences. (**A**–**F**) 1st–6th instar larvae; (**G**) Female adult (dorsal view); (**H**) Male adult (dorsal view); (**I**) Female pupa; (**J**) Male pupa; (**K**) Prepupa; (**L**) Female adult (ventral view); (**M**) Male adult (ventral view); (**N**) Eggs; (**O**) Larva feeding on a leaf; (**P**) Neighbor-Joining phylogenetic tree, The red text indicates the target Ascotis selenaria sample sequenced in this study.

The morphology of the different developmental stages of the moth is shown in Figure 1. Eggs were oval and pale green (Figure 1N). Larvae passed through six instars (Figure 1A–F) and displayed variable body coloration (dark brown, pale green, yellowish-brown, etc.). A pair of yellowish tubercles was present dorsally on the second abdominal segment, and a smaller pair of tubercles was on the eighth abdominal segment (evident after the fifth instar). Pupae were reddish-brown (Figure 1I,J). Adults (Figure 1G,H,L,M) were dark gray, with gray-black wavy lines on both fore- and hindwings, each featuring a gray-white stellate median spot. Female moths possessed filiform antennae, while males had pectinate antennae. All morphological characteristics were consistent with the description of *A. selenaria* [15].

The Neighbor-Joining tree (Figure 1P) was constructed using the sequence of the test sample (target *A*. *selenaria)* and all Ascotis sequences from the GenBank database. The figure indicates that the test sample sequence clustered with the genus Ascotis and showed the highest match (97.13%) with KX862852.1 in the database, confirming its classification within the genus Ascotis. The *A. selenaria* population collected from the eucalypt forest in Southern China showed the closest evolutionary relationship with the *A. selenaria* population from Pakistan. They form a distinct monophyletic clade supported by a strong bootstrap value of 96%, providing robust molecular evidence for their close genetic relationship and differentiation from other geographic populations. In contrast, its evolutionary relationship with other Chinese *A. selenaria* populations (including *A. selenaria* and the subspecies *A. selenaria dianaria*) recorded in GenBank was relatively distant, suggesting potential genetic differentiation in the Guangxi population infesting eucalyptus.

### 3.2. Biological Characteristics and Developmental Patterns

#### 3.2.1. Key Developmental Periods and Annual Life History

**Figure 2:**
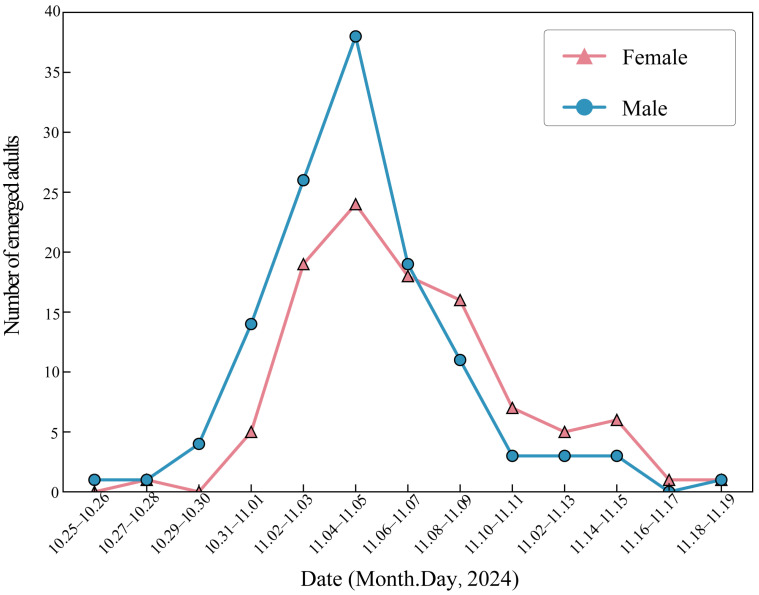
Adult emergence pattern of *A*. *selenaria* within one generation.

Under (26 ± 2) °C conditions, the average duration for *A. selenaria* to complete one generation (egg to adult emergence) was 52.29 days. The detailed developmental duration for each stage is summarized in Table 1. The egg stage lasted approximately 5.60 days, while the total larval stage (instars 1–6) was 27.52 days. The pre-pupal and pupal stages lasted 2.57 and 9.73 days, respectively. Significant sexual dimorphism was observed in developmental duration (Table 1). The male pupal stage was significantly longer than that of females (*t* = −3.64, *p* < 0.001). However, the emergence pattern (Figure 2) indicated that males emerged 1–2 days earlier than females (protandry). Adult longevity also differed significantly (*t* = −2.66, *p* = 0.009), with males living longer (approx. 7.3 days) than females (approx. 6.1 days).

#### 3.2.2. Larval Head Capsule Width, Body Length, and Feeding Volume by Instar

Larvae passed through six instars, and their morphological characteristics and feeding consumption are presented in Table 1. There were significant differences in head capsule width and body length among instars (*p* < 0.001). The growth of head capsule width and body length followed exponential patterns, with regression equations and high coefficients of determination (*R*^2^ > 0.99) presented in Table 1, adhering to Dyar’s Law. Feeding analysis revealed that food consumption increased significantly with larval development (Table 1). The 6th instar larvae exhibited the highest feeding capacity, consuming 3.459 g of leaves, which accounted for 79.68% of the total larval consumption. Collectively, the final three instars (4th–6th) were responsible for over 98% of the total food intake, highlighting the critical window for pest control prior to the 4th instar.

### 3.3. Adult Reproductive Biology

#### 3.3.1. Morphology of Male and Female Reproductive Systems

**Figure 3:**
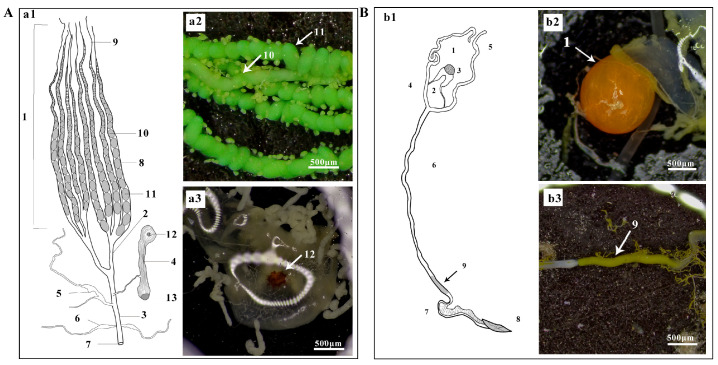
Reproductive systems of *A. selenaria*. (**A**) Female reproductive system: (**a1**) Schematic diagram; (**a2**,**a3**) Photographs of partial structures; (1) Ovary; (2) Lateral oviduct; (3) Common oviduct; (4) Bursa copulatrix; (5) Spermatheca; (6) Accessory gland; (7) Oviporus; (8) Ovariole; (9) Terminal filament; (10) Immature oocyte; (11) Mature egg; (12) Signum; (13) Ostium bursae. (**B**) Male reproductive system: (**b1**) Schematic diagram; (**b2**,**b3**) Photographs of partial structures; (1) Testis; (2) Vas deferens; (3) Seminal vesicle; (4) Duplex ejaculatory duct; (5) Accessory gland; (6) Simplex ejaculatory duct; (7) Cuticular segment of simplex; (8) Aedeagus; (9) Distal end of simplex ejaculatory duct.

The female reproductive system (Figure 3A) primarily consists of a pair of ovaries (with eight polytrophic ovarioles each), lateral oviducts, a common oviduct, a bursa copulatrix, a spermatheca, and accessory glands. The bursa copulatrix is characterized by a distinctive reddish-brown, circular, spined plate (signum). The male reproductive system (Figure 3B) includes a pair of fused, orange-yellow, spherical testes, vasa deferentia, seminal vesicles, accessory glands, and a single ejaculatory duct. The distal end of the single ejaculatory duct contains a characteristic golden-yellow fluid.

#### 3.3.2. Ovarian Development Progress in Female Moths

**Figure 4:**
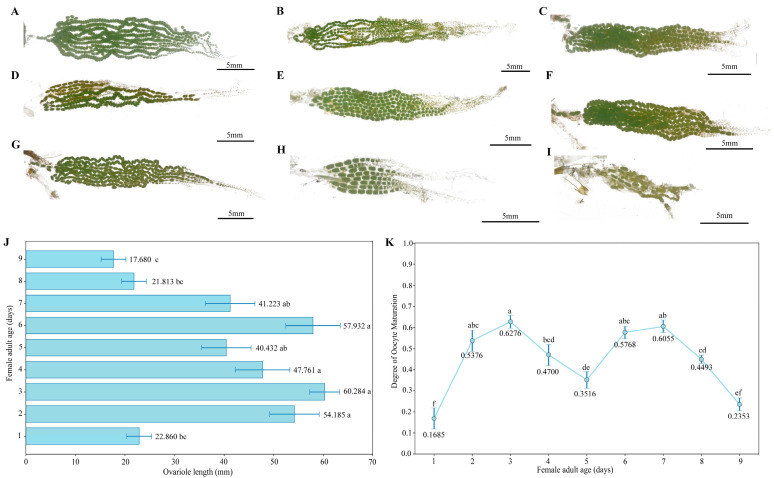
Ovarian development in female *A. selenaria*. (**A**–**I**) Ovarian morphology of females aged 1–9 days; (**J**) Ovariole length of females aged 1–9 days; (**K**) Degree of egg maturation in ovaries of females aged 1–9 days. Note: Different lowercase letters indicate significant differences in egg maturation between ages (*p* < 0.05) according to Duncan’s new multiple range test.

Ovarian development in female moths changed distinctly with adult age (Figure 4A–I). Ovariole length reached its maximum on day 3 (Figure 4J). The degree of egg maturation in the ovaries (Figure 4K) was very low on day 1. It then followed a bimodal trend of “increase–decrease–increase–decrease,” reaching maturation peaks on days 3 and 7, respectively. By day 9, almost all eggs had been laid.

#### 3.3.3. Changes in Male Internal Reproductive System with Age and Mating Status

**Figure 5:**
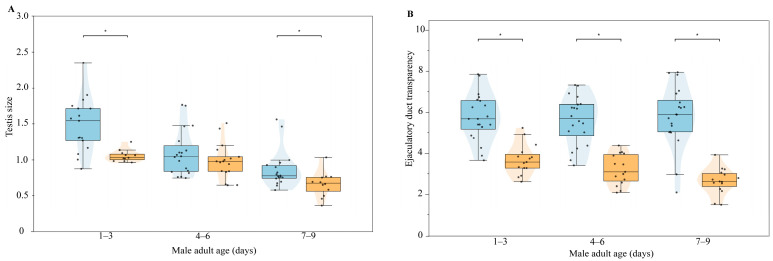
Changes in male reproductive organs with age and mating status. (**A**) Testis size of males at different ages; (**B**) Transparency of the distal end of the simplex ejaculatory duct under different ages and mating statuses. The asterisk (*) indicates a significant difference between groups (*p* < 0.05); Blue boxes indicate “before mating” status, and orange boxes indicate “after mating” status.

The male internal reproductive system was also significantly influenced by age and mating status. The testes of unmated males gradually decreased in size with increasing age (Figure 5A). The size of the testes before and after mating differed significantly in males aged 1–3 days and 7–9 days, with a notable reduction observed after mating (Supplementary Figure S1). Furthermore, the mating status had a highly significant impact on the transparency of the yellow fluid at the distal end of the single ejaculatory duct (Figure 5B), with the fluid becoming distinctly paler or transparent after mating (Supplementary Figure S2).

#### 3.3.4. Effect of Adult Age on Reproductive Capacity

The combined effects of female and male age on reproductive output are summarized in Table 2. Two-way ANOVA results indicated that egg production was primarily determined by female age (*F*_2,63_ = 29.62, *p* < 0.001), with younger females (1–3 days) laying significantly more eggs than older ones. The main effect of male age was also significant (*F*_2,63_ = 3.92, *p* = 0.025). The interaction between female and male age was marginally significant (*F*_4,63_ = 2.46, *p* = 0.055), suggesting a trend where the negative impact of delayed mating on fecundity was most pronounced when both partners were older.

Regarding egg hatchability, female age showed a highly significant main effect (*F*_2,27_ = 59.40, *p* < 0.001), with hatchability dropping sharply in females aged 7–9 days (Table 2). The main effect of male age was not statistically significant (*p* = 0.287). However, a marginally significant interaction was observed between female and male age (*F*_4,27_ = 2.41, *p* = 0.074). Biological trends indicated that while young males could maintain relatively higher hatchability (~54.6%) even when mated with old females (7–9 days), this capacity diminished with male aging, resulting in the lowest hatchability (~23.0%) in “old × old” (7–9 days × 7–9 days) pairings.

## 4. Discussion

This study systematically elucidated the biological characteristics of the eucalypt defoliator *A. selenaria* using a combination of molecular phylogenetics, morphology, and physiological ecology. It clarified the phylogenetic status of the Southern China population and, more importantly, revealed its unique developmental patterns, feeding mode, and reproductive strategy. These findings provide a solid theoretical basis and practical path for formulating scientific and precise Integrated Pest Management (IPM) strategies.

The molecular phylogenetic analysis based on the COI gene indicated that the *A. selenaria* population infesting eucalyptus in Guangxi is most closely related to the South Asian populations (India, Pakistan), forming an independent evolutionary clade, and exhibits significant genetic differentiation from the Northern China populations (e.g., Beijing). This pattern suggests that the population structure is not driven by isolation due to geographical distance but is profoundly influenced by large-scale climate and geographical barriers. While *A. selenaria* is highly polyphagous and known to feed on diverse hosts such as tea and citrus in other regions, our survey in Southern China has so far primarily detected outbreaks on Eucalyptus. However, given its broad host range, potential shifts to native vegetation or other economic crops in the region cannot be ruled out and warrant further monitoring. The genetic continuity between the Southern China and South Asian populations is likely maintained by the East Asian Summer Monsoon system, which forms a coherent subtropical–tropical moist ecological corridor favorable for gene flow between populations [17,18]. Conversely, the significant genetic divergence between the northern and southern populations reflects a biogeographical boundary created by the sharp climatic gradient from subtropical to temperate zones [19,20]. A similar pattern has been documented in *Sesamia inferens* [18], suggesting this is a common evolutionary pattern among Asian Lepidoptera.

Regarding larval development, this study confirmed that this population possesses six instars. While this differs from the five instars reported in some literature [21,22], it is typical of developmental plasticity, where environmental factors such as temperature, photoperiod, and food quality can significantly regulate instar number [23,24,25,26]. The “threshold size hypothesis” [27] provides the most plausible explanation for the six-instar phenomenon. Under conditions of relatively low nutritional quality of the food [28,29], a failure to reach the critical weight threshold before the fifth instar triggers a developmental checkpoint, leading to a sixth instar to complete the necessary biomass accumulation [28]. More critically, the feeding volume exhibited significant exponential growth. Instars 4–6 accounted for 98.32% of total consumption, with the 6th instar alone contributing 79.68%. This pattern is a classic “capital-breeding” strategy [30]—the larvae must accumulate sufficient energy during a short period of intense feeding to support the non-feeding pupal tissue reconstruction [31] and adult reproduction. Given the limited mobility of the larvae, which restricts effective behavioral thermoregulation [32], the visually apparent crown damage observed in the forest environment indicates that the crucial control window (98% of potential consumption) has already been missed. Therefore, the Economic Threshold (ET) must be reformulated, moving away from “visual damage assessment” and instead focusing on the monitoring of “low-density 1st–3rd instar larvae”. The paradigm established for the *Leucoptera coffeella* [33], should be adopted to shift IPM from a “reactive” to a “preventive” approach. This involves coordinating the precise application of selective biocontrol agents, guided by phenology models (e.g., accumulated degree-days [34]), to coincide with the early appearance of the larvae before the massive consumption phase [12].

The adult reproductive strategy in this study presents two key adaptations. First, significant protandry was observed, with males emerging 1–2 days earlier than females. Second, the reproductive senescence was highly asymmetrical: female mating delay (ages 1–9 days) led to a sharp decline in oviposition rate and hatchability. This decline was modulated by a marginally significant interaction effect between male and female age (*p* = 0.074). We found that the influence of male age was not negligible but highly context-dependent. For young females (1–3 days), male age had virtually no effect on hatchability. However, for old females (7–9 days), the ‘old age’ of the male (7–9 days) further exacerbated the collapse of female reproductive capacity, particularly egg hatchability. Protandry is recognized as a classic evolutionarily stable strategy (ESS) [35,36]: males maximize mating opportunities by emerging early [37,38,39], and females reduce predation risk by shortening “mate-searching time” [40], thereby avoiding the high cost of delayed mating [36]. The dramatic decline in female fecundity with delayed mating is a phenomenon confirmed across various Lepidoptera species [41,42,43], physiologically mediated by the rapid senescence and oosorption of unfertilized oocytes [44]. These two phenomena form a clear causal chain: females face intense “time constraint” [36], and protandry is the evolutionary response to this pressure, ensuring that females can mate immediately upon emergence to prevent reproductive collapse.

A particularly novel finding in this study is the bimodal pattern of female ovarian maturation with age (peaks on days 3 and 7), which is distinctly different from the typical continuous maturation observed in Lepidoptera [45]. We propose the hypothesis of “reproductive bet-hedging” [46,47,48]: to explain this pattern. In this context, bet-hedging refers to a strategy where females distribute their reproductive effort over time to minimize the risk of total reproductive failure in an unpredictable environment. The first peak (day 3) represents an immediate investment in “current reproduction,” a response to the urgent physiological constraint of reproductive senescence. If oviposition fails due to ecological risks (e.g., lack of host plant, high predation, no male encounter), the female enters a developmental “plateau phase” on days 4–6 to conserve resources. The second peak (day 7) is interpreted as an insurance investment in “future reproduction,” an adaptive trade-off that embodies the principle of “Do not put all your eggs in one basket” [46]. This strategy maximizes geometric mean fitness in unpredictable environments, offering a new perspective for understanding the diversity of insect reproductive strategies.

Collectively, these findings point to two high-leverage IPM intervention windows. The first is the “Preventive Larval Window,” which, based on the late-instar feeding voracity (98.32% of consumption by instars 4–6), necessitates moving control efforts forward to the 1st–3rd instars. The second is the “Disruptive Adult Window,” based on protandry, the high cost of mating delay for females, and the newly discovered bimodal ovarian maturation rhythm (first peak on day 3). Regarding protandry, this phenomenon provides a strategic advantage for the deployment of Mating Disruption (MD). Since males emerge 1–2 days earlier to maximize mating opportunities [36], they are active and searching before females appear. Deploying synthetic sex pheromones (female-related attraction cues) prior to the onset of male emergence is crucial. By saturating the environment with these cues early, the first-emerging males are desensitized or confused immediately upon emergence, effectively “blinding” them before they can locate the later-emerging females [49].

Furthermore, the novel bimodal pattern of ovarian maturation (peaking on days 3 and 7) raises the question of whether sex pheromone production follows a similar rhythm. Previous research on other Lepidoptera suggests a tight physiological correlation between ovarian development and pheromone biosynthesis, ensuring that calling behavior coincides with peak fertility [50,51]. If *A. selenaria* exhibits synchronized pheromone pulses corresponding to these ovarian peaks, the MD strategy must maintain high efficacy specifically during these two physiological windows to prevent mating when females are most fertile.

We recommend that Mating Disruption (MD) devices using sex pheromones must be deployed before the emergence of the first males. This action is intended to “blind” the earliest emerging males, preventing females from mating within their first reproductive peak (day 3) and consequently leading to the physiological failure of their unfertilized eggs [52,53]. This dual-window, phenology-driven, precise IPM strategy [12] is both scientifically sound and practically feasible, offering an environmentally friendly path for the sustainable management of eucalyptus.

## 5. Conclusions

This study provides a comprehensive profile of A. selenaria on Eucalyptus, confirming six larval instars and revealing exponential feeding by the final instar (79.68% of total consumption), necessitating a shift in the control focus to the early larval window (1st–3rd instars). Adult reproductive biology is characterized by protandry and extreme female sensitivity to mating delay, which, coupled with a novel bimodal ovarian maturation pattern (peaks at days 3 and 7), define a narrow window of reproductive vulnerability. These findings establish a dual-window Integrated Pest Management (IPM) framework: combining phenology-guided biocontrol for early instars with pre-male-flight deployment of Mating Disruption to target the adult reproductive asymmetry. However, it remains unclear whether the second peak of ovarian maturation is accompanied by a second wave of sex pheromone release. Future research should investigate the synchronization between ovarian development and calling behavior to determine if mating disruption protocols need to be extended to cover this late-stage reproductive window. This life-history-based strategy offers a sustainable, science-driven pathway for managing this emerging defoliator in subtropical plantations.

## Figures and Tables

**Table 1:** Life history parameters, morphological growth models, and feeding consumption of *A. selenaria*.

Developmental Stage	Instar/Sex	Sample Size (*n*)	Duration (Days)	Head Capsule Width (mm)	Body Length (mm)	Feeding Volume (g) (% of Total)
Egg	—	1022	5.60 ± 0.221	—	—	—
Larva	1st Instar	15	3.60 ± 0.400 b	0.29 ± 0.004 f	2.90 ± 0.076 f	0.006 (0.14%) c
	2nd Instar	13	6.08 ± 0.702 a	0.46 ± 0.011 e	4.27 ± 0.070 e	0.017 (0.39%) c
	3rd Instar	13	3.85 ± 0.478 b	0.72 ± 0.012 d	7.08 ± 0.183 d	0.040 (0.92%) c
	4th Instar	14	3.07 ± 0.245 b	1.26 ± 0.046 c	13.53 ± 0.546 c	0.229 (5.28%) bc
	5th Instar	15	3.78 ± 0.236 b	2.10 ± 0.047 b	23.22 ± 0.429 b	0.590 (13.59%) b
	6th Instar	14	7.14 ± 0.376 a	3.33 ± 0.055 a	39.81 ± 0.826 a	3.459 (79.68%) a
Prepupa	—	14	2.57 ± 0.137	—	—	—
Pupa	Female	104	9.57 ± 0.885 b	—	—	—
	Male	124	9.97 ± 0.775 a	—	—	—
Adult	Female	59	6.14 ± 2.063 b	—	—	—
	Male	105	7.29 ± 2.931 a	—	—	—
Statistics	*F*/*t*-value	—	F_5,78_ = 14.74	*F*_5,78_ = 1033.67	*F*_5,78_ = 985.01	*F*_5,78_ = 118.25
	*p*-value	—	*p* < 0.001	*p* < 0.001	*p* < 0.001	*p* < 0.001
Growth Model	Equation	—	—	y = 0.1721e^0.4948x^	y = 1.5457e^0.5379x^	—
	*R* ^2^	—	—	0.9989	0.9958	—

Note: 1. Data are presented as Mean ± SE (Standard Error) for larval duration and morphological traits, and Mean for feeding volume. 2. Values within the same column followed by different lowercase letters indicate significant differences based on Duncan’s new multiple range test (*p* < 0.05). 3. Statistical Analysis Results: (1) Larval Instars (One-way ANOVA): Significant differences were observed in duration (*F*_5,78_ = 14.74, *p* < 0.001), head capsule width (*F*_5,78_ = 1033.67, *p* < 0.001), body length (*F*_5,78_ = 985.01, *p* < 0.001), and feeding volume (*F*_5,78_ = 118.25, *p* < 0.001). (2) Pupa (*t*-test): Significant difference in duration between sexes (*t* = −3.64, *df* = 226, *p* < 0.001). (3) Adult (*t*-test): Significant difference in longevity between sexes (*t* = −2.66, *df* = 162, *p* = 0.009). Growth models describe the exponential relationship between larval instar (x) and morphological metrics (y).

**Table 2:** Effects of female and male adult age on the fecundity and hatchability of *A. selenaria*.

Female Age (Days)	Male Age (Days)	Egg Production	Egg Hatchability (%)
1–3	1–3	632.92 ± 58.30	88.00 ± 3.45
	4–6	458.50 ± 56.59	92.37 ± 1.84
	7–9	317.67 ± 16.05	91.31 ± 1.71
4–6	1–3	361.25 ± 48.46	80.65 ± 2.39
	4–6	422.80 ± 72.93	80.41 ± 9.60
	7–9	382.75 ± 60.31	79.25 ± 7.14
7–9	1–3	152.50 ± 37.77	54.58 ± 0.87
	4–6	121.67 ± 50.24	37.38 ± 5.16
	7–9	58.33 ± 20.19	23.02 ± 7.11
Two-way ANOVA		F-value (*p*-value)	F-value (*p*-value)
Source of Variation			
Female Age (F)		*F*_2,63_ = 29.62 (*p* < 0.001)	*F*_2,27_ = 59.40 (*p* < 0.001)
Male Age (M)		*F*_2,63_ = 3.92 (*p* = 0.025)	*F*_2,27_ = 1.31 (*p* = 0.287)
Interaction (F × M)		*F*_4,63_ = 2.46 (*p* = 0.055)	*F*_4,27_ = 2.41 (*p* = 0.074)

Note: Data are presented as Mean ± SE. 1. *p*-values indicate significance levels from Two-way ANOVA. 2. Egg hatchability data were analyzed as proportions (0–1) but presented as percentages for clarity.

## Data Availability

The datasets used and/or analyzed in the current study are availablefrom the corresponding author on reasonable request. The data are not publicly available as the data in this study provide support for future laboratory experiments.

## Supplementary Materials

The following supporting information can be downloaded at: https://www.mdpi.com/article/10.3390/biology14121780/s1, Figure S1: Testes of males at different ages and mating statuses; Figure S2: Distal end of the simplex ejaculatory duct.
