# Supplementary material for: Life History of the Giant Looper Moth Ascotis selenaria (Lepidoptera: Geometridae) in Eucalyptus Plantations and the Effect of Adult Mating Age on Fecundity
Source: Biology (Basel). 2025 Dec 13;14(12):1780. doi: 10.3390/biology14121780 (PMC12730433; doi:10.3390/biology14121780)
Supplement: Supplementary file 1 [file biology-14-01780-s001.zip › Figure Legends.pdf]

Figure S1. Testes of males at different ages and mating statuses. (A) 1–3 days pre-mating, (B) 4–6 days pre-mating, (C) 7–9 days pre-mating, (D) 1–3 days post-mating, (E) 4–6 days post-mating, (F) 7–9 days post-mating.

Figure S2. Distal end of the simplex ejaculatory duct. (A) 1–3 days pre-mating, (B) 4–6 days pre-mating, (C) 7–9 days pre-mating, (D) 1–3 days post-mating, (E) 4–6 days post-mating, (F) 7–9 days post-mating.
