# Supplementary figures and images for: Life History of the Giant Looper Moth Ascotis selenaria (Lepidoptera: Geometridae) in Eucalyptus Plantations and the Effect of Adult Mating Age on Fecundity
Source: Biology (Basel). 2025 Dec 13;14(12):1780. doi: 10.3390/biology14121780 (PMC12730433; doi:10.3390/biology14121780)

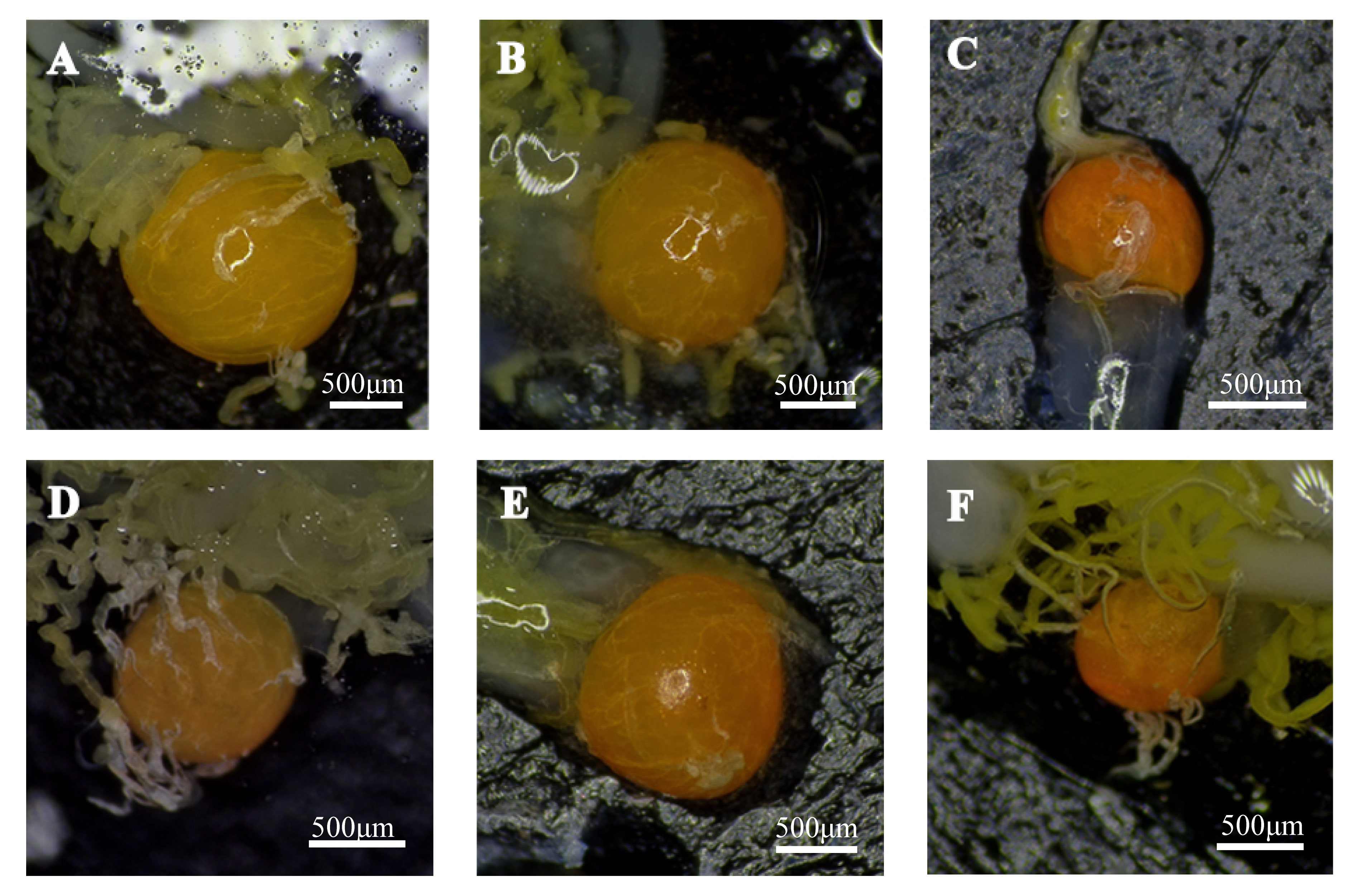

Supplement: Supplementary file 1 [file biology-14-01780-s001.zip › Figure S1.png]

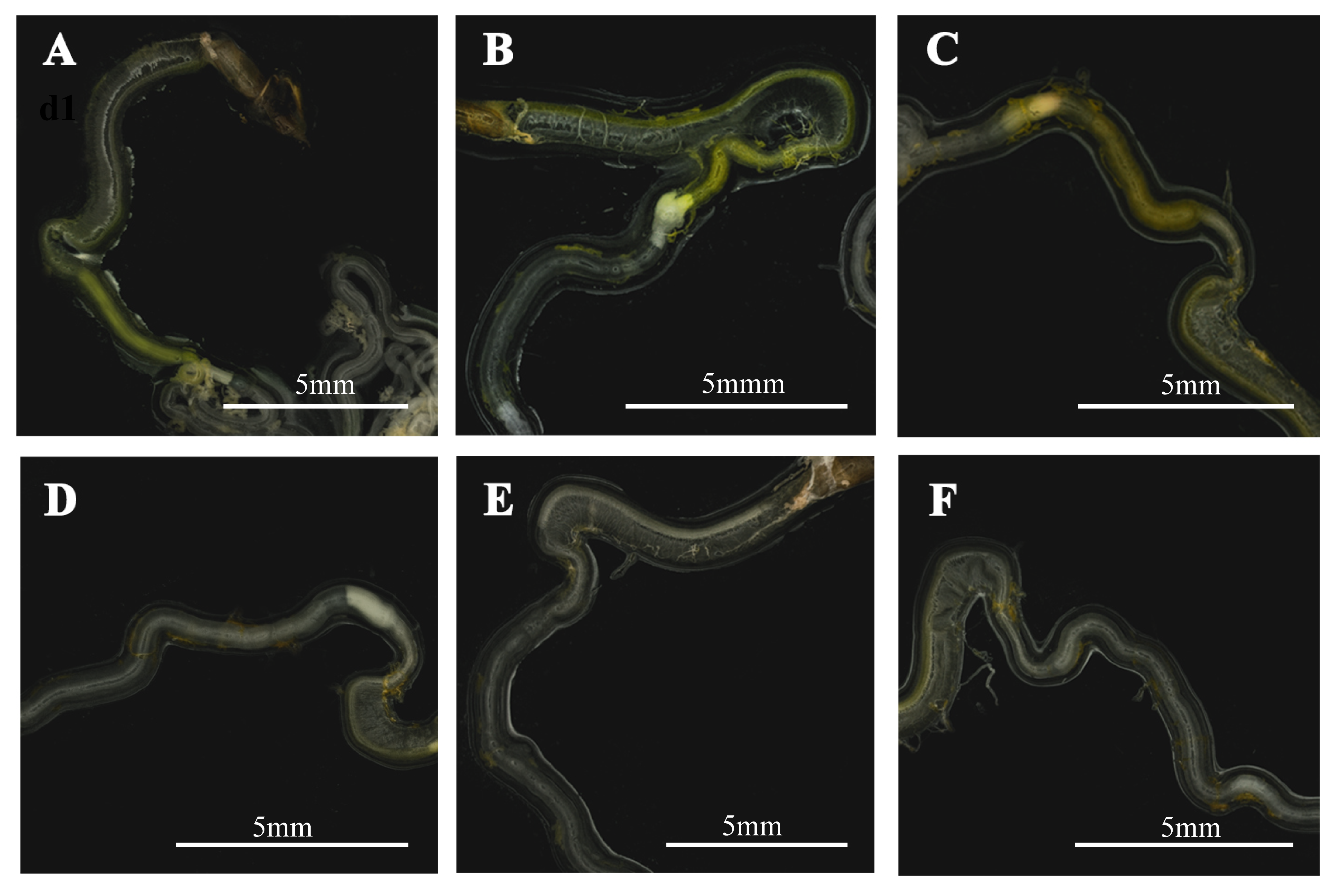

Supplement: Supplementary file 1 [file biology-14-01780-s001.zip › Figure S2.png]
